# Differences in epidemiological and clinical features between adult and pediatric tracheobronchial tuberculosis patients in Southwest China

**DOI:** 10.3389/fpubh.2023.1225267

**Published:** 2023-07-19

**Authors:** Qing Chen, Tao Huang, Liping Zou, Liangshuang Jiang, Jiying Sun, Xiaoli Lu, Xiaoqiu Huang, Guihui Wu, Wei He

**Affiliations:** ^1^Department of Tuberculosis, Public Health Clinical Center of Chengdu, Chengdu, China; ^2^Department of Thoracic Surgery, Public Health Clinical Center of Chengdu, Chengdu, China; ^3^Department of Health Information, Public Health Clinical Center of Chengdu, Chengdu, China

**Keywords:** tracheobronchial tuberculosis, pediatric, children, adult, epidemiology

## Abstract

**Background:**

Tracheobronchial tuberculosis (TBTB) is a common form of extrapulmonary tuberculosis that affects the tracheobronchial tree. However, the mechanism has not been fully elucidated. Comparisons of clinical characteristics in various age groups can aid in the understanding of TBTB.

**Methods:**

This retrospective study was conducted at the Public Health Clinical Center of Chengdu between July 2017 and December 2021, including adults and children with TBTB. Clinical data were extracted from medical records. T/T' test, Mann-Whitney U test, Chi-square test, or Fisher's exact test were used in this study.

**Results:**

This study enrolled 347 patients with TBTB (175 adults and 172 children). Adult females were more susceptible to TBTB, whereas gender-based differences were not observed in children. Children had a higher occurrence of irritant dry cough and fever, and acute hematogenous disseminated PTB, and specific types of EPTB, but a shorter interval before diagnosis, and lower diagnostic yields compared to adults (*P* < 0.05). Adults presented more extensive lung lesions and cavitations as compared to children. Granulation hyperplasia and lymph fistula were more frequently observed in children, as well as airway stenosis, but less severe.

**Conclusions:**

The study revealed important variations exist in multiple respects between adults and children with TBTB.

## 1. Introduction

Prior to the emergence of COVID-19, tuberculosis (TB) was among the leading causes of death resulting from infectious diseases. In 2021, an estimated 10.6 million individuals worldwide developed TB, with children (under 15 years of age) accounting for 11% of cases (1). In 2020, China carried 7.4% of the global TB burden (1). Notably, there are regional variations in TB incidence within China, with the western region exhibiting higher rates than the eastern and central regions (2). Sichuan Province, situated in Southwest China, is home to the Chengdu Public Health Clinical Center (PHCC), which is a designated hospital for tuberculosis. Patients suffering from TB from neighboring provinces such as Guizhou, Yunnan, and Tibet often seek treatment at PHCC, making the TB patients at PHCC representative of the Southwest China region.

Tracheobronchial tuberculosis (TBTB) is a form of TB that affects the tracheobronchial tree. Despite its prevalence, the pathological process of TBTB has not been fully understood. Studies have shown that 6–50% of patients with pulmonary tuberculosis (PTB) also have TBTB (3–7). In China, TBTB is the primary cause of benign airway stenosis. However, the exact pathogenesis of TBTB is still unclear and there is limited evidence on the relationship between age and clinical features of TBTB. To our knowledge, differences in epidemiological and clinical characteristics between children and adults with TBTB have not been previously investigated. This study aims to describe the differences in TBTB epidemiological and clinical characteristics between adults and children, examine the relationship between age and TBTB clinical characteristics, and provide insights for clinical decision-making or investigation of TBTB immune mechanism.

## 2. Methods

### 2.1. Study design and participants

This study is a hospital-based cross-sectional study including inpatients who were recruited from PHCC (Chengdu, China) between July 2017 and December 2021. According to the guidelines recommended by the Chinese Medical Association (8), all patients were bacteriologically or clinically diagnosed as TBTB as shown in the case definition section. This includes adults (≥18 years) and children ( ≤ 14 years) who underwent bronchoscopy on admission and were diagnosed with TBTB. We excluded patients with viral infections, those who had undergone bronchoscopy before admission, or those with incomplete medical records.

### 2.2. Case definition of PTB and TBTB

This study adopted the WHO PTB diagnostic criteria (1). Currently, there is no gold standard for the diagnosis of TBTB in the world. Therefore, the diagnosis of TBTB cases was made by qualified physicians according to the guideline for diagnosis and treatment of TBTB in China (8) for reference, (1) Clinical symptoms of TB, especially irritable cough, expectoration, hemoptysis or dyspnea; (2) Acid-fast bacilli (AFB) positive, mycobacterium PCR or Gene Xpert MTB/RIF assay (Xpert) positive in a sputum smear, brush smear or bronchial alveolar lavage fluid (BALF); (3) Mtb culture positive; (4) bronchoscopy positive; and (5) bronchoscopic biopsy. In 2000, Chung's study divided TBTB into seven subtypes by bronchoscopy: actively caseating, edematous-hyperemic, fibrostenotic, tumorous, granular, ulcerative, and nonspecific bronchitic (3). However, the subtype of TBTB in China is different from Chung's and is adopted in this study. Subtypes of TBTB (8): (1) Inflammatory infiltration; (2) Ulceration necrosis; (3) Granulation hyperplasia; (4) Cicatrices stricture; (5) Tracheobronchialmalacia; (6) Lymph fistula. Moreover, bronchoscopic appearances of each subtype can be referred to published study (9).

### 2.3. Data collection

Epidemiological, demographic and clinical data were extracted from electronic medical records. Baseline laboratory tests and bronchoscopy were performed on admission. All data were reviewed by two physicians (QC and TH), and a third researcher (WH) assessed whether there were differences in interpretation between the two lead reviewers.

### 2.4. Laboratory examination

All patients were tested for human immunodeficency virus (HIV) testing (HIV DNA PCR if < 18 months, HIV antibody if >18months) (10). Respiratory samples including sputum and BALF were tested at PHCC. Samples were detected by smear microscopy for AFB and Gene Xpert, and pretreated for purification and homogenization according to WHO laboratory standard guidelines (11), and then BACTEC MGIT960 system (Becton Dickinson & Co., Franklin Lakes, NJ, USA) was used for MTB culture.

### 2.5. Bronchoscopy examination

All patients underwent flexible bronchoscopy with BALF on admission. TBTB type as described in the *Case definition of PTB and TBTB* and lesion areas were collected. The grades of obstruction were described as previously reported: mild (< 50%), moderate (50–75%), or severe (>75%) (12). BALF samples obtained were sent for smear microscopy, Xpert, and mycobacterial liquid culture.

### 2.6. Statistical analysis

Continuous variables that conformed to a normal distribution were expressed as mean ± standard deviation, and variables that were not normally distributed were expressed as the median. Categorical variables were expressed as *n*(%). T/T' test, Mann-Whitney U test, Chi-square test or Fisher's exact test were used appropriately to compare the differences between pediatric and adult TBTB patients. A two-tailed *p* < 0.05 was considered statistically significant. All statistical analyzes were performed using SPSS version 21.0 (SPSS Inc, Chicago, IL, USA).

## 3. Results

### 3.1. Patient characteristics

From July 2017 to December 2021, Chengdu PHCC admitted 347 TBTB inpatients including 175 adults (50.4%) and 172 children (49.6%), with 157 males (45.2%) and 190 females (54.8%). The median age of all patients was 17 years, range 1–67 years. There were 326 (93.9%) patients who came from suburbs or rural. Compared with adults, children in suburban or rural areas had a higher proportion of TBTB (97.1% vs. 90.9%, *P* = 0.015). Adult TBTB patients had a lower proportion of BCG vaccination compared with children (4.0% vs. 14.5%, *P* = 0.001). More than half of pediatric TBTB patients (52.3%) had primary TB, whereas almost all adult TBTB patients (99.4%) had secondary TB. The proportions of acute hematogenous disseminated PTB and most EPTB (tuberculous pleurisy, extrathoracic tuberculous lymphadenitis, tuberculous peritonitis, tuberculous meningitis) in children with TBTB were higher than those in adults (all *P* < 0.05). The incidences of pneumonia, anemia, hypertension, and diabetes in adult TBTB patients were higher than those in children (all *P* < 0.05). Only two adults were co-infected with HIV. The clinical presentation of TBTB was nonspecific. Children with TBTB were more likely to have dry cough and fever, while adults had a higher proportion of dyspnea and hemoptysis (all *P* < 0.001). The time from symptom onset to diagnosis of TBTB was longer in adults compared with children (*P* < 0.001). There was no significant difference between adults and children with TBTB in terms of sex and TB history (*P* > 0.05). Demographic and clinical characteristics of adult and pediatric TBTB patients are presented in Table 1.

**Table 1:** Demographic and clinical characteristics of adult and pediatric TBTB patients.

**Characteristics**	**Total (*n* = 347)**	**Adults (*n =* 175)**	**Children (*n =* 172)**	***χ^2^/Z***	***P value***
**Age, years**	17.00 (12.00, 32.00)	31.00 (24.00, 46.00)	12.00 (9.00, 13.00)	*Z* = −16.129	< 0.001
**Sex**				2.398	0.121
Female	190 (54.8)	103 (58.9)	87 (50.6)		
Male	157 (45.2)	72 (41.1)	85 (49.4)		
**Residence**				5.933	0.015
Urban	21 (6.1)	16 (9.1)	5 (2.9)		
Suburb or rural	326 (93.9)	159 (90.9)	167 (97.1)		
**TB history**	97 (28.0)	50 (28.6)	47 (27.3)	0.067	0.796
**BCG history**	32 (9.2)	7 (4.0)	25 (14.5)	11.500	0.001
**Type of PTB**					
Primary PTB	91 (26.2)	1 (0.6)	90 (52.3)	120.089	< 0.001
Secondary PTB	256 (73.8)	174 (99.4)	82 (47.7)	120.089	< 0.001
Acute hematogenous disseminated PTB	17 (4.9)	4 (2.3)	13 (7.6)	5.176	0.023
Subacute hematogenous disseminated PTB	3 (0.9)	0 (0)	3 (1.7)	1.380^a^	0.240
**EPTB**					
Tuberculous pleurisy	139 (40.1)	59 (33.7)	80 (46.5)	5.916	0.015
Extrathoracic tuberculous lymphadenitis	154 (44.4)	34 (19.4)	120 (69.8)	89.048	< 0.001
Tuberculous peritonitis	52 (15.0)	14 (8.0)	38 (22.1)	13.523	< 0.001
Tuberculous pericarditis	54 (15.6)	27 (15.4)	27 (15.7)	1.714	0.190
Tuberculous meningitis	23 (6.6)	3 (1.7)	20 (11.6)	13.775	< 0.001
Osseous tuberculosis	8 (2.3)	3 (1.7)	5 (2.9)	0.146^a^	0.702
Tuberculosis of genitourinary system	3 (0.9)	1 (0.6)	2 (1.2)	0.000^a^	0.988
**Comorbidity**					
Pneumonia	133 (38.3)	77 (44.0)	56 (32.6)	4.804	0.028
Anemia	62 (17.9)	40 (22.9)	22 (12.8)	5.990	0.014
Hypoproteinemia	44 (12.7)	19 (10.9)	25 (14.5)	1.060	0.303
Diabets	18 (5.2)	18 (10.3)	0 (0)	18.659	< 0.001
Hypertension	9 (2.6)	9 (5.1)	0 (0)	7.160	0.007
HIV	2 (0.6)	2 (1.1)	0 (0)	0.486^a^	0.486
**Clinical symptoms**					
Cough	301 (86.7)	149 (85.1)	152 (88.4)	0.787	0.375
Sputum	193 (55.6)	116 (66.3)	77 (44.8)	16.272	< 0.001
Fever (temperature ≥37.3°C)	127 (36.6)	41 (23.4)	86 (50.0)	26.394	< 0.001
Weight reduction	71 (20.5)	34 (19.4)	37 (21.5)	0.231	0.631
Dyspnea	41 (11.8)	33 (18.9)	8 (4.7)	16.801	< 0.001
Chest pain	48 (13.8)	27 (15.4)	21 (12.2)	0.754	0.385
Hemoptysis	29 (8.4)	26 (14.9)	3 (1.7)	19.475	< 0.001
Night Sweats	7 (2.0)	3 (1.7)	4 (2.3)	0.001	0.982
Hoarseness	3 (0.9)	1 (0.6)	2 (1.2)	0.000^a^	0.988
**Time from symptoms onset to diagnosis of TBTB**	64.00 (25.00, 180.00)	120.00 (34.00, 240.00)	36.00 (20.25, 95.75)	*Z* = −5.339	< 0.001

PTB, pulmonary tuberculosis; EPTB, extrapulmonary tuberculosis; TBTB, tracheobronchial tuberculosis.

^a^Continuous correction of chi-square.

### 3.2. Bacteriological examination outcomes and yields of laboratory methods for TBTB diagnosis

The results of Mtb bacteriological examination outcomes and rifampicin resistance are presented in Table 2. Only 13.4% of children with TBTB had positive sputum smear. The diagnostic yields of smear/culture/Gene Xpert in sputum and BALF were significantly higher in adults with TBTB than those in children (*P* < 0.05), respectively. Among 102 adult TBTB patients with negative sputum smears (of whom 61 had negative BALF smear), the diagnostic yields of sputum culture and Gene Xpert were 26.5% (27/102) and 41.2% (42/102), respectively, and the diagnostic rates of BALF were 46.1% (47/102) and 55.9% (57/102). In 46 patients with negative sputum and BALF culture, the diagnostic yields of Gene Xpert with both samples were 10.9% (5/46) and 39.1% (18/46), respectively (data not shown). Among 149 TBTB children with negative sputum smears, the diagnostic yields of sputum culture and Gene Xpert were 22.1% (33/149) and 35.6% (53/149), and in BALF were 25.5% (38/149) and 51.7% (77/149), respectively. Among the 134 TBTB children with negative sputum and BALF, the diagnostic yields of Gene Xpert with sputum and BALF were 32.8% (44/134) and 46.3% (62/134), respectively. Moreover, in 97 patients with negative sputum and BALF culture, the diagnostic yields of Gene Xpert were 26.8% (26/97) and 39.2% (38/97), respectively (data not shown). Adult TBTB patients had a higher proportion of rifampicin gene resistance compared with children (*P* < 0.01).

**Table 2:** Bacteriological examination outcomes of adult and pediatric TBTB patients.

**Laboratory examinations**	**Total (*n* = 347)**	**Adults (*n* =175)**	**Children (*n* = 172)**	**χ^2^**	***P* value**
**Sputum test results**					
Smear-positivity	96/347 (27.7)	73/175 (41.7)	23/172 (13.4)	34.819	< 0.001
Culture-positivity	158/347 (45.5)	106/175 (60.6)	52/172 (30.2)	32.194	< 0.001
Gene Xpert MTB/RIF-positivity	187/347 (53.9)	115/175 (65.7)	72/172 (41.9)	19.863	< 0.001
Rifampicin-resistant in Xpert	55/347 (15.9)	51/175 (29.1)	4/172 (2.3)	46.771	< 0.001
**BALF test results**		..			
Smear-positivity	89/347 (25.6)	61/175 (34.9)	28/172 (16.3)	15.700	< 0.001
Culture-positivity	163/347 (47.0)	108/175 (61.7)	55/172 (32.0)	30.796	< 0.001
Gene Xpert MTB/RIF-positivity	245/347 (70.6)	133/175 (76.0)	112/172 (65.1)	4.951	0.026
Rifampicin-resistant in Xpert	64/347 (18.4)	55/175 (31.4)	9/172 (5.2)	39.573	< 0.001

TBTB, tracheobronchial tuberculosis; BALF, bronchial alveolar lavage fluid.

In addition, Gene Xpert in BALF had the highest diagnostic yield (70.6%) (*P* < 0.05) (Table 3). Furthermore, the diagnostic yields of sputum culture (45.5%) and sputum Gene Xpert (53.9%) were significantly higher than sputum smear (27.7%) (*P* < 0.05). Whereas, the diagnostic yields of BALF culture (47.0%) were significantly higher than BALF smear (25.6%) (*P* < 0.05).

**Table 3:** Comparison of diagnostic yields of different laboratory methods.

**Method A vs. B**	**Diagnostic yield (%/totals)**		**χ^2^**	***P* value**
	**Method A**	**Method B**		
Sputum smear vs. sputum culture	96/347 (27.7)	158/347 (45.5)	23.870	< 0.001
Sputum smear vs. Gene Xpert in sputum	96/347 (27.7)	187/347 (53.9)	49.410	< 0.001
Sputum culture vs. Gene Xpert in sputum	158/347 (45.5)	187/347 (53.9)	4.847	0.028
BALF smear vs. BALF culture	89/347 (25.6)	163/347 (47.0)	46.436	< 0.001
BALF smear vs. Gene Xpert in BALF	89/347 (25.6)	245/347 (70.6)	140.462	< 0.001
BALF culture vs. Gene Xpert in BALF	163/347 (47.0)	245/347 (70.6)	39.991	< 0.001
Sputum smear vs. BALF smear	96/347 (27.7)	89/347 (25.6)	0.361	0.548
Sputum culture vs. BALF culture	96/347 (27.7)	163/347 (47.0)	27.652	< 0.001
Gene Xpert in sputum vs. Gene Xpert in BALF	187/347 (53.9)	245/347 (70.6)	20.627	< 0.001

BALF, bronchial alveolar lavage fluid.

### 3.3. Imaging features

The CT findings of adult and pediatric TBTB patients are shown in Table 4. Among them, atelectasis accounted for 13.5%, and 36.6% had cavitary lesions. Lesions involving unilateral lung and multiple lobes were more common (75.4%) in adult TBTB patients compared to children (*P* < 0.05). There were no significant differences in other TBTB-related imaging features (including airway stenosis, obstructive pneumonia, and roughness of bronchial wall) between adults and children (*P* > 0.05).

**Table 4:** CT imaging features of adult and pediatric TBTB patients in current study.

**Characteristics**	**Total (*n =* 347)**	**Adults (*n =* 175)**	**Children (*n =* 172)**	**χ^2^**	***P* value**
Pulmonary atelectasis	47 (13.5)	20 (11.4)	27 (15.7)	1.350	0.245
Airway stenosis	56 (16.1)	27 (15.4)	29 (16.9)	0.131	0.717
Obstructive pneumonia	16 (4.6)	9 (5.1)	7 (4.1)	0.227	0.634
Roughness of bronchial wall	51 (14.7)	27 (15.4)	24 (14.0)	0.151	0.698
Pulmonary cavities	127 (36.6)	88 (50.3)	39 (22.7)	28.500	< 0.001
Lesions involve the lung fields				66.305	< 0.001
Unilateral lung, single lobe	43 (12.4)	6 (3.4)	37 (21.5)		
Unilateral lung, multiple lobes	100 (28.8)	30 (17.1)	70 (40.7)		
Bilateral lungs, single lobe	13 (3.7)	7 (4.0)	6 (3.5)		
Bilateral lungs, multiple lobes	191 (55.0)	132 (75.4)	59 (34.3)		

TBTB, tracheobronchial tuberculosis.

### 3.4. Bronchoscopic examination

The results of bronchoscopy in TBTB adults and children are shown in Table 5. Ulceration necrosis (30.3%) and granulation hyperplasia (39.0%) were the most common subtypes in adults and children with TBTB, respectively. The granulation hyperplasia and lymph fistula subtypes were more common in children with TBTB than in adults (49.0% vs. 14.9%, 21.2% vs. 0%, *P* < 0.001). Adults had more inflammatory infiltration and cicatrices stricture subtypes compared with children (29.7% vs. 18.0%, 25.1% vs. 14.5%, *P* < 0.05). Mtb often affects the upper lobe bronchi bilaterally. The left upper lobe bronchus (36.9%) was more affected than the other bronchi in this study. Seventy-nine point five percent of the total had tracheobronchial stenosis. Tracheobronchial stenosis was more severe in children with TBTB than in adults (84.3% vs. 74.9%, *P* < 0.05).

**Table 5:** Bronchoscopic examination characteristics of adult and pediatric TBTB patients.

**Characteristics**	**Total (*n =* 347)**	**Adults (*n =* 175)**	**Children (*n =* 172)**	**χ^2^**	***P* value**
**Subtypes of TBTB**				87.659^a^	< 0.001
**Inflammatory infiltration**	83 (23.9)	52 (29.7)	31 (18.0)		
Ulceration necrosis	72 (20.7)	53 (30.3)	19 (11.0)		
Granulation hyperplasia	93 (26.8)	26 (14.9)	67 (39.0)		
Cicatrices stricture	69 (19.9)	44 (25.1)	25 (14.5)		
Tracheobronchial malacia	1 (0.3)	0 (0)	1 (0.6)		
Lymph fistula	29 (8.4)	0 (0)	29 (21.2)		
**Site of lesions**					
Trachea	20 (5.8)	13 (7.4)	7 (4.1)	1.802	0.180
Right main bronchus	50 (14.4)	23 (13.1)	27 (15.7)	0.459	0.498
Right upper lobe bronchus	118 (34.0)	69 (39.4)	49 (28.5)	4.626	0.031
Right middle bronchus	54 (15.6)	25 (14.3)	29 (16.9)	0.351	0.553
Right middle lobe bronchus	50 (14.4)	21 (12.0)	29 (16.9)	1.662	0.197
Right lower lobe bronchus	52 (15.0)	27 (15.4)	25 (14.5)	0.131	0.717
Left main bronchus	76 (21.9)	39 (22.3)	37 (21.5)	0.030	0.862
Left upper lobe bronchus	128 (36.9)	62 (35.4)	66 (38.4)	0.323	0.570
Left lower lobe bronchus	77 (22.2)	39 (22.3)	38 (22.1)	0.002	0.966
**Number of lesions involved**				0.003	0.959
Single	176 (50.7)	89 (50.9)	87 (50.6)		
Multiple	171 (49.3)	86 (49.1)	85 (49.4)		
**Tracheobronchial stenosis**	276 (79.5)	131 (74.9)	145 (84.3)	27.046	< 0.001
Mild	79 (22.8)	18 (10.3)	61 (35.5)		
Moderate	114 (32.9)	65 (37.1)	49 (28.5)		
Severe	83 (23.9)	48 (27.4)	35 (20.3)		
**Tracheobronchial obstruction**	42 (12.1)	18 (10.3)	14 (8.1)	0.477	0.490

TBTB, tracheobronchial tuberculosis.

^a^Likelyhood ratio of chi-square.

## 4. Discussion

This study is the first to investigate differences in the epidemiological and clinical characteristics of TBTB between adults and children in Southwest China. This study was conducted at Chengdu PHCC, a major TB hospital with TBTB patients radiating the whole southwest region of China, including but not limited to Sichuan, Tibet, Yunnan and Guizhou. The relative risk for PTB in the western region were significantly higher than those in the eastern and central regions (2). Therefore, TBTB patients in Chengdu PHCC may represent the Southwest region to some extent.

In the present study, adult females appeared to be more susceptible to TBTB, consistent with previous researches (13, 14) showing gender is a risk factor for developing TBTB. One possible explanation for the greater susceptibility of adult women to TBTB may be related with the relatively narrow airways and the habit of not coughing, which leads to the retention of Mtb-carrying sputum in the lumen. Furthermore, this may be related to female-specific differences in endocrine status and immune response that require further study. In the current study, it was observed that children with TBTB did not show any gender-based differences in susceptibility, the possible reason may be due to minimal sex differences among children. The current study found that up to 90% of adults and children with TBTB were from suburb or rural areas. The reason may be related to factors such as limited medical resources, limited economic capacity, and low education level in these areas, which lead to low BCG vaccination rate (90.8% in this study), delay in consultation time, missed diagnosis, and misdiagnosis. The incidences of acute hematogenous disseminated PTB and some types of EPTB in children with TBTB were higher than those in adults, which may reflect that children's immunity is lower than that of adults. In children with TBTB, irritant dry cough and fever were found to be more common, whereas adults with TBTB were more likely to experience sputum production, dyspnea, and hemoptysis. However, the underlying reasons for these differences between age groups require further exploration.There may be several reasons for the longer time from symptom onset to diagnosis in adults with TBTB compared with children. Adults have a higher tolerance for respiratory symptoms, and adults in suburban and rural areas may delay seeking healthcare for financial reasons. Children who develop symptoms at school, especially in groups, were taken to the hospital for PPD, interferon-gamma release assay (IGRA), AFB, and chest X-ray. Besides, it cannot be ruled out that the course or mechanism of TBTB in children differs from that in adults (immune response, etc.), leading to a more rapid progression of symptoms and earlier treatment in children.

This study showed that the diagnostic yields of Xpert from sputum and BALF specimens in both adults and children with TBTB were significantly higher than other detection methods (*P* < 0.05), suggesting that Xpert is the most effective initial diagnostic tool for TBTB patients. The diagnostic rate of AFB in adult TBTB sputum specimens was 41.7%, consistent with previous researches (0–53%) (4, 7, 15). However, the diagnostic yields of AFB and culture from sputum or BALF samples were lower in children with TBTB than in adults, suggesting that achieving bacteriological confirmation is more challenging in children with TBTB than in adults. The diagnostic rates of Xpert for TBTB children with negative sputum and BALF were 32.8% and 46.3%, respectively, indicating that Xpert has obvious advantages over traditional methods such as AFB and Mtb culture in TBTB children. Nevertheless, it needs to be taken into account that Xpert is expensive and has limited applicability in economically under developed countries.

In general, imaging examinations are very helpful in the diagnosis of PTB, but there are certain deficiencies in the identification of TBTB, unless there is airway obstruction, manifested as direct or indirect signs, such as atelectasis, airway stenosis, obstructive pneumonia and roughness of bronchial wall, otherwise imaging examination may not be able to detect endobronchial lesions, and the diagnostic accuracy is much lower than that of bronchoscopy. The incidences of atelectasis in children (11.4%) and adults (15.7%) with TBTB in this study were similar. Adult TBTB patients had more extensive lung lesions and more cavities than children, which is considered to be related to more secondary pulmonary tuberculosis in adults and longer course of TBTB leading to farther spread of lesions in the airway and more serious illness.

There are obvious differences between adults and children in TBTB subtypes in this study. The main subtypes in adults with TBTB were inflammatory infiltration (29.7%), ulceration necrosis (30.3%), and cicatrices stricture (25.1%), similar to the previous study (9). However, children with TBTB presented more granulation hyperplasia and lymph fistula than adults in this study. In the previous study, according to the classification of Chung et al. (3), tumorous type (92.4%) was the most common subtype among 157 children with endobronchial tuberculosis (16), and tumorous type may be equivalent to granulation hyperplasia and lymph fistula in China (8). Primary tuberculosis-based lymph fistula is the possible reason for the high proportion of lymph fistula subtype, while the high proportion of granulation-proliferating in children with TBTB is more likely to be related to the difference in immunity between adults and children, which needs to be further explored.

In this study, most of the lesions on bronchoscopy involved the upper lobe bronchi of the lung, and the proportion of lesions penetrating the left main bronchus was higher than that of the right main bronchus (21.9% vs. 14.4%). The possible reason is that the upper lobes of both lungs are prone to secondary pulmonary tuberculosis. The angle between the left main bronchus and the trachea is relatively large, and the lumen is slender, which is easily squeezed by the aortic arch and esophagus, making it easier to retain bacterial secretions, resulting in difficult drainage of the left bronchus and making the lesion more likely to invade the left main bronchus. The incidence of TBTB involving the right upper lobe bronchus in children was lower than that in adults, but the etiology is still unknown and further investigation is needed. There was no significant difference in the area involved in TBTB lesions between adults and children (*P* > 0.05).

Tracheobronchial stenosis is the most common complication of TBTB (17, 18), which may lead to poor drainage, obstructive pneumonia, atelectasis, etc. The incidence of tracheobronchial stenosis is about 20–91% (4, 9, 13, 14). In this study, the overall incidence of tracheobronchial stenosis was 79.5%, and the possible reason is that more patients had bronchoscopy by passive screening instead of active screening, similar to the previous research (9). Notably, children with TBTB had a higher incidence of airway stenosis compared with adults (84.3% vs. 74.9%), but less severe, which may partially explain why adults had more severe respiratory symptoms such as dyspnea. The possible reason may be that children have narrower airways and are more prone to stenosis, but they received bronchoscopy and medical care earlier than adults, so the stenosis was less severe than that of adults in this study.

In summary, we found that adult females appeared to be more susceptible to TBTB while children with TBTB had gender evenly. Up to 90% of TBTB patients came from suburb or rural areas. The incidences of acute hematogenous disseminated PTB and some types of EPTB, irritant dry cough, and fever were higher in children than in adults, while expectoration, dyspnea, and hemoptysis were more common in adults.The time from symptom onset to diagnosis in adults with TBTB was longer than in children. Xpert is the most effective initial diagnostic tool for TBTB patients. The diagnostic yields of AFB and culture from sputum or BALF samples were lower in children than in adults. Adult TBTB patients had more extensive lung lesions and more cavitation than children. Granulation hyperplasia and lymph fistula occurred more frequently in children than in adults. Compared with adults, children with TBTB had a higher incidence of airway stenosis, but the severity was less severe.

The current study still has several limitations. First, the study is only a retrospective study with a relatively small sample size. Second, this study is a single-center study, and there is a possibility of bias. Moreover, pulmonary function data were not available for this study, according to TBTB patients had a risk of transmission by aerosol, and pulmonary function tests were not routinely performed. Furthermore, assessing differences between adults and children with TBTB requires larger samples and multi-center studies with longer follow-ups after diagnosis.

## 5. Conclusion

In this study, we compared differences in epidemiological and clinical features between adults and children with TBTB in Southwest China. There are differences in gender distribution, clinical symptoms, EPTB, comorbidities, types of TBTB, lesions involving the lungs and bronchi, and airway stenosis in adults and children with TBTB. In addition, Long-term follow-up is needed in the future to further explore the influence of age on the treatment and prognosis of patients with TBTB.

## Data availability statement

The raw data supporting the conclusions of this article will be made available by the authors, without undue reservation.

## Ethics statement

The studies involving human participants were reviewed and approved by Human Ethics Committee of Public Health Clinical Center of Chengdu. Written informed consent to participate in this study was provided by the participants' legal guardian/next of kin.

## Author contributions

QC and WH had the idea for and designed the study and had full access to all of the data in the study and take responsibility for the integrity of the data and the accuracy of the data analysis. QC, TH, WH, and GW drafted the paper. QC, TH, WH, LZ, LJ, and GW did the analysis. QC, JS, XL, and XH collected the data. All authors have read and approved the final manuscript.
